# Epigenome-wide meta-analysis of blood DNA methylation and its association with subcortical volumes: findings from the ENIGMA Epigenetics Working Group

**DOI:** 10.1038/s41380-019-0605-z

**Published:** 2019-12-06

**Authors:** Tianye Jia, Congying Chu, Yun Liu, Jenny van Dongen, Evangelos Papastergios, Nicola J. Armstrong, Mark E. Bastin, Tania Carrillo-Roa, Anouk den Braber, Mathew Harris, Rick Jansen, Jingyu Liu, Michelle Luciano, Anil P. S. Ori, Roberto Roiz Santiañez, Barbara Ruggeri, Daniil Sarkisyan, Jean Shin, Kim Sungeun, Diana Tordesillas Gutiérrez, Dennis van’t Ent, David Ames, Eric Artiges, Georgy Bakalkin, Tobias Banaschewski, Arun L. W. Bokde, Henry Brodaty, Uli Bromberg, Rachel Brouwer, Christian Büchel, Erin Burke Quinlan, Wiepke Cahn, Greig I. de Zubicaray, Stefan Ehrlich, Tomas J. Ekström, Herta Flor, Juliane H. Fröhner, Vincent Frouin, Hugh Garavan, Penny Gowland, Andreas Heinz, Jacqueline Hoare, Bernd Ittermann, Neda Jahanshad, Jiyang Jiang, John B. Kwok, Nicholas G. Martin, Jean-Luc Martinot, Karen A. Mather, Katie L. McMahon, Allan F. McRae, Frauke Nees, Dimitri Papadopoulos Orfanos, Tomáš Paus, Luise Poustka, Philipp G. Sämann, Peter R. Schofield, Michael N. Smolka, Dan J. Stein, Lachlan T. Strike, Jalmar Teeuw, Anbupalam Thalamuthu, Julian Trollor, Henrik Walter, Joanna M. Wardlaw, Wei Wen, Robert Whelan, Liana G. Apostolova, Elisabeth B. Binder, Dorret I. Boomsma, Vince Calhoun, Benedicto Crespo-Facorro, Ian J. Deary, Hilleke Hulshoff Pol, Roel A. Ophoff, Zdenka Pausova, Perminder S. Sachdev, Andrew Saykin, Margaret J. Wright, Paul M. Thompson, Gunter Schumann, Sylvane Desrivières

**Affiliations:** 1 Social, Genetic and Developmental Psychiatry Centre, Institute of Psychiatry, Psychology & Neuroscience, King’s College London, London, UK; 2 Institute of Science and Technology for Brain-Inspired Intelligence, Fudan University, Shanghai, China; 3 MOE Key Laboratory of Computational Neuroscience and Brain-Inspired Intelligence, Fudan University, Shanghai, China; 4 MOE Key Laboratory of Metabolism and Molecular Medicine, Department of Biochemistry and Molecular Biology, School of Basic Medical Sciences, Zhongshan Hospital, Fudan University, Shanghai, China; 5 Vrije Universiteit, Amsterdam, Dept Biological Psychology, Van der Boechorststraat 1, 1081 BT Amsterdam, The Netherlands; 6 Mathematics and Statistics, Murdoch University, Perth, WA Australia; 7 Brain Research Imaging Centre, Centre for Clinical Brain Sciences, and Centre for Cognitive Ageing and Cognitive Epidemiology, University of Edinburgh (MEB), Edinburgh, UK; 8 Department of Translational Research in Psychiatry, Max-Planck Institute of Psychiatry, Kraepelinstr, 2-10 80804 Munich, Germany; 9 Centre for Clinical Brain Sciences and Edinburgh Imaging, University of Edinburgh, Edinburgh, UK; 10 Department of Psychiatry, VU University Medical Centre, Amsterdam, The Netherlands; 11 Department of Electrical Engineering, University of New Mexico, Albuquerque, NM USA; 12 Centre for Cognitive Ageing and Cognitive Epidemiology, Department of Psychology, University of Edinburgh, Edinburgh, UK; 13 UCLA Center for Neurobehavioral Genetics, University of California, Los Angeles, Los Angeles, CA USA; 14 Department of Psychiatry, University Hospital Marqués de Valdecilla, School of Medicine, University of Cantabria, Santander, Spain; 15 Centro Investigación Biomédica en Red de Salud Mental, Santander, Spain; 16 Box 591, Uppsala biomedicinska centrum BMC, Husarg. 3, 751 24, Uppsala, Sweden; 17 Hospital for Sick Children, University of Toronto, Toronto, ON Canada; 18 Center for Neuroimaging, Department of Radiology and Imaging Sciences, Indiana University School of Medicine, Indianapolis, IN USA; 19 Center for Computational Biology and Bioinformatics, Indiana University School of Medicine, Indianapolis, IN USA; 20 Neuroimaging Unit, Technological Facilities. Valdecilla Biomedical Research Institute IDIVAL, Santander, Cantabria Spain; 21 National Ageing Research Institute, Parkville, VIC Australia; 22 Academic Unit for Psychiatry of Old Age, University of Melbourne, St George’s Hospital, Kew, VIC Australia; 23 Institut National de la Santé et de la Recherche Médicale, INSERM Unit 1000 “Neuroimaging & Psychiatry”, University Paris Sud-Paris Saclay, University Paris Descartes, Orsay, France; 24 DIGITEO Labs, Gif sur Yvette, France; 25 GH Nord Essonne Psychiatry Department 91G16, Orsay, France; 26 Department of Child and Adolescent Psychiatry and Psychotherapy, Central Institute of Mental Health, Medical Faculty Mannheim, Heidelberg University, Square J5, 68159 Mannheim, Germany; 27 Discipline of Psychiatry, School of Medicine and Trinity College Institute of Neuroscience, Trinity College Dublin, Dublin, Ireland; 28 Centre for Healthy Brain Ageing, School of Psychiatry, University of New South Wales, Sydney, NSW Australia; 29 Dementia Centre for Research Collaboration, School of Psychiatry, University of New South Wales, Sydney, NSW Australia; 30 University Medical Centre Hamburg-Eppendorf, House W34, 3.OG, Martinistr. 52, 20246 Hamburg, Germany; 31 Department of Psychiatry and Brain Center Rudolf Magnus, University Medical Center Utrecht, Utrecht, The Netherlands; 32 Faculty of Health, Institute of Health and Biomedical Innovation, Queensland University of Technology, Brisbane, QLD Australia; 33 Division of Psychological and Social Medicine and Developmental Neurosciences, Faculty of Medicine, TU Dresden, Germany; 34 Department of Clinical Neuroscience, Karolinska Institutet, Center for Molecular Medicine, Karolinska University Hospital, Stockholm, Sweden; 35 Department of Cognitive and Clinical Neuroscience, Central Institute of Mental Health, Medical Faculty Mannheim, Heidelberg University, Square J5, Mannheim, Germany; 36 Department of Psychology, School of Social Sciences, University of Mannheim, 68131 Mannheim, Germany; 37 Department of Psychiatry and Neuroimaging Center, Technische Universität Dresden, Dresden, Germany; 38 NeuroSpin, CEA, Université Paris-Saclay, F-91191 Gif-sur-Yvette, France; 39 Departments of Psychiatry and Psychology, University of Vermont, 05405 Burlington, VT USA; 40 Sir Peter Mansfield Imaging Centre School of Physics and Astronomy, University of Nottingham, University Park, Nottingham, UK; 41 Charité—Universitätsmedizin Berlin, corporate member of Freie Universität Berlin, Humboldt-Universität zu Berlin, Berlin, Germany; 42 Berlin Institute of Health, Department of Psychiatry and Psychotherapy, Campus Charité Mitte, Charitéplatz 1, Berlin, Germany; 43 Department of Psychiatry and Neuroscience Institute, University of Cape Town, Cape Town, South Africa; 44 Physikalisch-Technische Bundesanstalt (PTB), Braunschweig and Berlin, Berlin, Germany; 45 Imaging Genetics Center, Mark and Mary Stevens Neuroimaging & Informatics Institute, Keck School of Medicine of the University of Southern California, Marina del Rey, CA USA; 46 Central Clinical School—Brain and Mind Centre, The University of Sydney, Camperdown, NSW 2050 Australia; 47 School of Medical Sciences, University of New South Wales, Sydney, NSW Australia; 48 Genetic Epidemiology, QIMR Berghofer Medical Research Institute, Brisbane, QLD Australia; 49 Maison de Solenn, Cochin Hospital, Paris, France; 50 Neuroscience Research Australia, Sydney, NSW Australia; 51 Herston Imaging Research Facility, School of Clinical Sciences, Queensland University of Technology, Brisbane, QLD Australia; 52 Institute for Molecular Bioscience, University of Queensland, Brisbane, QLD Australia; 53 Bloorview Research Institute, Holland Bloorview Kids Rehabilitation Hospital and Departments of Psychology and Psychiatry, University of Toronto, Toronto, ON M6A 2E1 Canada; 54 Department of Child and Adolescent Psychiatry and Psychotherapy, University Medical Centre Göttingen, von-Siebold-Str. 5, 37075 Göttingen, Germany; 55 Faculty of Medicine, University of New South Wales, Sydney, Australia; 56 SAMRC Unit on Risk & Resilience in Mental Disorders, Cape Town, South Africa; 57 Queensland Brain Institute, University of Queensland, Brisbane, QLD Australia; 58 Department of Developmental Disability Neuropsychiatry, School of Psychiatry, University of New South Wales, Sydney, NSW Australia; 59 Brain Research Imaging Centre, Centre for Clinical Brain Sciences, Edinburgh Dementia Research Centre, and Centre for Cognitive Ageing and Cognitive Epidemiology, University of Edinburgh, Edinburgh, UK; 60 UK Dementia Research Institute at the University of Edinburgh, Edinburgh, UK; 61 School of Psychology and Global Brain Health Institute, Trinity College Dublin, Dublin, Ireland; 62 Department of Neurology, Indiana University School of Medicine, Indianapolis, IN USA; 63 Department of Radiology and Imaging Sciences, Indiana University School of Medicine, Indianapolis, IN USA; 64 Department of Medical and Molecular Genetics, Indiana University School of Medicine, Indianapolis, IN USA; 65 Indiana Alzheimer Disease Center, Indiana University School of Medicine, Indianapolis, IN USA; 66 Tri-institutional Center for Translational Research in Neuroimaging and Data Science (TReNDS), Emory University, 30303, Atlanta, GA USA; 67 Semel Institute for Neuroscience and Human Behavior, University of California, Los Angeles, Los Angeles, CA USA; 68 Neuropsychiatric Institute, Prince of Wales Hospital, Sydney, NSW Australia; 69 Radiology and Imaging Sciences, Indiana University School of Medicine, Indianapolis, IN 46202 USA

**Keywords:** Genetics, Molecular biology, Neuroscience

## Abstract

DNA methylation, which is modulated by both genetic factors and environmental exposures, may offer a unique opportunity to discover novel biomarkers of disease-related brain phenotypes, even when measured in other tissues than brain, such as blood. A few studies of small sample sizes have revealed associations between blood DNA methylation and neuropsychopathology, however, large-scale epigenome-wide association studies (EWAS) are needed to investigate the utility of DNA methylation profiling as a peripheral marker for the brain. Here, in an analysis of eleven international cohorts, totalling 3337 individuals, we report epigenome-wide meta-analyses of blood DNA methylation with volumes of the hippocampus, thalamus and nucleus accumbens (NAcc)—three subcortical regions selected for their associations with disease and heritability and volumetric variability. Analyses of individual CpGs revealed genome-wide significant associations with hippocampal volume at two loci. No significant associations were found for analyses of thalamus and nucleus accumbens volumes. Cluster-based analyses revealed additional differentially methylated regions (DMRs) associated with hippocampal volume. DNA methylation at these loci affected expression of proximal genes involved in learning and memory, stem cell maintenance and differentiation, fatty acid metabolism and type-2 diabetes. These DNA methylation marks, their interaction with genetic variants and their impact on gene expression offer new insights into the relationship between epigenetic variation and brain structure and may provide the basis for biomarker discovery in neurodegeneration and neuropsychiatric conditions.

## Introduction

Structural brain measures are important correlates of developmental and health outcomes across the lifetime. A large body of evidence has revealed age-related reductions in grey matter structures across the brain [1], notably in the hippocampus, which correlates with declining memory performance in older adults [2, 3]. Recent findings from large-scale neuroimaging analyses within the ENIGMA consortium have revealed consistent patterns of cortical [4, 5] and subcortical [5–8] brain volume reductions across several neuropsychiatric disorders. Of all structures reported, the hippocampus was the most consistently and robustly altered, being smaller in major depressive disorder [6], schizophrenia [7], attention deficit hyperactivity disorder [8], obsessive-compulsive disorder (OCD) [9], and posttraumatic stress disorder [10]. Other notable changes included volume reductions in the thalamus and nucleus accumbens (NAcc) in schizophrenia [7, 8], as well as volume increases in the same regions in paediatric OCD [9].

Such differences in brain structure may fundamentally reflect the effects of genetic and environmental factors and their interplay, as suggested by the study of discordant monozygotic twins [11]. DNA methylation is an epigenetic mechanism that may underlie gene–environment contributions to brain structure. It is under the influence of genetic [12, 13] and developmental [13–15] factors and plays an important role in brain development and disease, by regulating gene expression. DNA methylation is also a mechanism through which external stimuli, such as the environment, may contribute to expression of common diseases such as neurodegenerative disorders [16].

While efforts to identify genetic factors influencing brain structure have flourished in recent years [17–19], epigenetic studies of brain-related phenotypes remain very sparse. A considerable constraint is the need for a surrogate tissue for epigenetic studies of the living human brain. Crucially, while initial reports have demonstrated that although DNA methylation patterns are largely tissue-specific, often differing between blood and brain [20, 21], there are also similarities [22] and blood DNA methylation shows promise as a biomarker for brain-related traits, including neuropsychiatric disorders [23–27], cognitive ability [28, 29] and future psychopathology [26]. However, only a few studies of small sample sizes have reported associations between blood DNA methylation and brain phenotypes [26, 30–32].

Here, we built upon these findings and performed a large multisite epigenome-wide association study (EWAS) of structural brain volumes in 3337 individuals from 11 cohorts. We focussed on analyses of the hippocampus, thalamus and NAcc, based on relevance of these subcortical brain regions for disease and on heritability of these phenotypes. We selected the hippocampus as the brain structure most consistently and robustly altered in neuropsychiatric disorders, as described above. We also selected the thalamus and NAcc as subcortical regions with the highest and lowest twin-based heritability estimates, respectively [18, 33], to test a model according to which a substantial fraction of the heritability of complex traits may be due to epigenetic variation [34].

## Material and methods

### Subjects and brain measures

The brain phenotypes examined in this study are from the ENIGMA analysis of high-resolution MRI brain scans of volumetric measures (full details in [18]). Our analyses were focussed to mean (of left and right hemisphere) volumetric measures of three subcortical areas: the hippocampus, thalamus and NAcc, selected for their link to disease, different levels of heritability, and developmental trajectories. MRI brain scans and genome-wide DNA methylation data were available for 3337 subjects from 11 cohorts (Supplementary Table 1). All participants in all cohorts in this study gave written informed consent and sites involved obtained approval from local research ethics committees or Institutional Review Boards.

### DNA methylation microarray processing and normalization

Blood DNA methylation was assessed for each study using the Illumina HumanMethylation450 (450k) microarray, which measures CpG methylation across >485,000 probes covering 99% of RefSeq gene promoters [35], following the manufacturer’s protocols. Standardised quality control procedures and quantile normalization were performed using the *minfi* Bioconductor package in R [36]. Please refer to Supplementary Materials and Methods for more details.

### Epigenome-wide association analysis

EWAS with volumes of the thalamus, hippocampus and NAcc were performed for each site separately with standardised procedures (see Supplementary Materials and Methods for details), where control variables included sex, age, age [2], intracranial volume, methylation composition (the first four components), blood cell-type composition (the first two components), sample batches (when applicable), recruitment centres (when applicable) and disease status (when applicable). For studies with data collected across several centres, dummy-coded covariates were also included in the model.

Results from each cohort were then meta-analysed by combining correlations (fisher’s r-to-Z transformed) across all 11 cohorts with a fixed effect model, weighted by the corresponding inversed variance [37]. False discovery rates (FDR) were computed (correcting for the number of brain regions tested and the number of DNA methylation probes) and FDR < 0.05 was considered statistically significant. Differentially methylated regions (DMRs) were identified by applying the Comb-p algorithm [38] (Supplementary Materials and Methods).

## Results

### Associations of DNA methylation with subcortical volumes: analyses of individual CpG sites

**Fig. 1:**
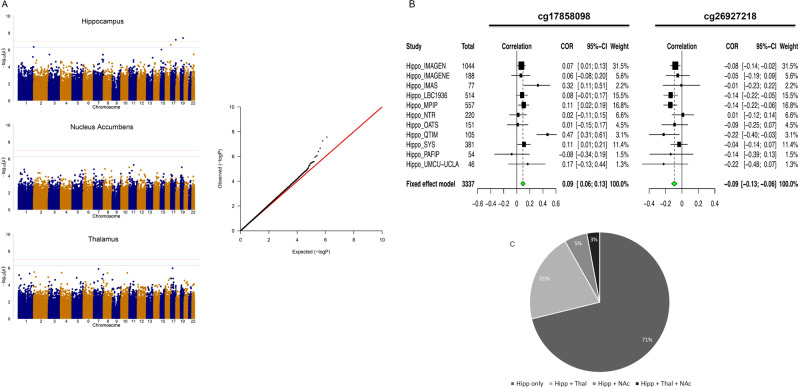
**a** Manhattan plots (*left*) summarizing the association results for the hippocampus, thalamus and NAcc volumes. The red and blue lines represent the genome-wide FDR significance level (corrected for three brain regions) and non-corrected FDR significance level, respectively. Quantile–quantile plots (*right*) of multivariate GWAS of all traits (volumes of the hippocampus, thalamus and accumbens) show that the observed *P* values only deviate from the expected null distribution at the most significant values, indicating no undue inflation of the results. **b** Forest plots show the effect (i.e. correlations between CpG methylation and hippocampus volume) at each of the contributing sites to the meta-analysis. The size of the dot is proportional to the sample size, the correlation level is shown on the *x*-axis, and confidence interval is represented by the line. **c** Pie chart of distribution of the 340 CpGs associated with hippocampus volume at *P* < 5 × 10^−4^. The chart indicates the proportion of these CpG sites that are unique to the hippocampus or that are also associated (nominally, at *p* < 0.05) with the two other volumetric phenotypes investigated. In general, CpGs that influence other phenotypes than hippocampus volume have higher effect on thalamus than on NAcc volume

We first investigated the association of DNA methylation at individual CpG sites in whole blood samples with the mean bilateral volumes of the hippocampus, thalamus and NAcc. Meta-analysis was applied by combining correlations across all 11 cohorts with a fixed effect model, weighting for sample size. We identified two CpGs associating with the volume of the hippocampus (Fig. 1a; Supplementary Table 2) at an experiment-wide (correcting for the number of brain regions tested) FDR < 0.05. The analyses of thalamus and NAcc volumes identified no CpG reaching the experiment-wide FDR threshold. Q–Q plots for the *P* values of the analyses showed no evidence of *P* value inflation. The CpGs associated with hippocampal volume explained each 0.9% of the phenotypic variance. Their effects were consistent across cohorts, with similar effect sizes for the cg26927218 site (*P* > 0.1, Cochran’s *Q* test), while moderate heterogeneity in the magnitude, but not the direction of effects was noted for cg17858098 (Fig. 1b). Effect sizes for analyses with and without patients across the 11 cohorts were very highly correlated (*r* ≥ 0.99) for CpGs with *P* < 1 × 10^−3^, indicating that these effects were unlikely driven by disease. These CpGs were annotated to the brain-specific angiogenesis inhibitor 1-associated protein 2 (*BAIAP2*) gene (also known as *IRSp53*; cg26927218)—encoding a synaptic protein whose expression in the hippocampus is required for learning, memory [39] and social competence [40] and to the enoyl-CoA hydratase-1 (*ECH1*; cg17858098), which encodes an enzyme involved in the β-oxidation of fatty acids [41].

CpGs associated with hippocampal volume showed effects specific for this structure rather than pleiotropic effects. Of the 340 CpGs associated with hippocampus volume at *P* < 5 × 10^−4^ (Supplementary Table 2), 71% were associated only with the hippocampus, 21% were shared with the thalamus and few with the NAcc (Fig. 1c). These closer epigenetic links between hippocampus and thalamus reflected closer correlations between their volumes (*r*_H*T_ = 0.367, *P* = 5.78 × 10^−34^ and *r*_H*N_ = 0.201, *P* = 8.36 × 10^−11^, for correlations of hippocampal volumes with thalamus and NAcc volumes, respectively).

### Associations of DNA methylation with subcortical volumes: differentially methylated regions

The analyses described above did not account for effects of DNA methylation clusters at regions formed by spatially correlated CpGs, which often occur within regulatory regions in the genome and are powerful means to control gene expression. Therefore, in the following analyses, we set out to identify such DNA methylation clusters (i.e. differentially methylated regions, DMRs) by applying the *comb-p* algorithm [38] to our epigenome-wide meta-analyses of hippocampal volume (see Supplementary Materials and Methods). Several DMRs significantly associated with the volume of hippocampus in the meta-analysed results (Šidák [42] corrected *P* < 0.05, number of consecutive probes ≥E2; total numbers of DMRs = 20; Table 1). A DMR that included the cg26927218 site was identified (*P*_corrected_ = 9.44 × 10^−4^), further supporting the association of *BAIAP2* methylation with hippocampal volume. In addition to being identified from the meta-analysed data, three of these DMRs were identified in at least two cohorts, when analyses were run on EWAS results of each cohort separately, indicating that their association with brain volumes were unlikely to be due to chance. They were located within the cardiomyopathy associated gene 5 (*CMYA5*; *P*_corrected_ = 8.47 × 10^−14^; this DMR is subsequently referred to as DMR1), encoding an expression biomarker for diseases affecting striated muscle [43–46] and possibly a schizophrenia risk gene [47]; the hematopoietically expressed homeobox (*HHEX*; *P*_corrected_ = 9.27 × 10^−5^; DMR2) gene, encoding a homeobox transcription factor controlling stem cells pluripotency and differentiation in several tissues [48–52], and a well-known risk loci for type 2 diabetes [53], as well as the carnitine palmitoyltransferase 1B (*CPT1B*; *P*_corrected_ = 2.45 × 10^−4^; DMR3) gene, encoding a rate-limiting enzyme in the mitochondrial beta-oxidation of long-chain fatty acids, whose expression enhances reprogramming of somatic cells to induced pluripotent stem cells [54], cancer cell self-renewal and chemoresistance [55]. There was a significant degree of correlation of DNA methylation at these DMRs (*r* = 0.155, *P* = 7.30 × 10^−8^ and *r* = 0.147, *P* = 2.91 × 10^−7^, for DMR1 versus DMR3 and DMR1 versus DMR2, respectively). These three DMRs were also taken forward for further analyses.

**Table 1:** List of DMRs identified from the the hippocampus EWAS meta-analysis results

Chrom no.	Start	End	n_probes	z_p	z_sidak_p	Nearest gene	Distance to TSS	Other gene	Distance to TSS	Number of cohorts individually identifying the DMR
**Hippocampus**										
**chr5**	**78,985,425**	**78,985,593**	**9**	**3.39E−17**	**8.47E−14**	**CMYA5**	**−191**			**3**
chr7	149,569,715	149,570,184	12	4.34E−08	3.88E−05	ATP6V0E2	−107			1
**chr10**	**94,455,543**	**94,455,896**	**4**	**7.79E−08**	**9.27E−05**	**HHEX**	**7775**	**EXOC6**	**−152,557**	**2**
chr14	23,623,480	23,623,936	6	1.24E−07	1.14E−04	CEBPE	−34,883	SLC7A8	29,141	0
**chr22**	**51,016,501**	**51,016,900**	**7**	**2.33E−07**	**2.45E−04**	**CPT1B**	**395**			**2**
chr3	130,745,442	130,745,686	10	4.63E−07	7.97E−04	NEK11	−163	ASTE1	82	0
chr2	161,504,772	161,504,906	3	2.65E−07	8.30E−04	TANK	−511,969	RBMS1	−154,534	0
chr7	130,626,376	130,626,560	3	3.83E−07	8.73E−04	MKLN1	−386,151	KLF14	−207,580	0
chr17	79,053,905	79,054,074	3	3.80E−07	9.44E−04	BAIAP2	45,028	AATK	85,827	0
chr8	66,472,662	66,472,956	3	9.33E−07	1.33E−03	CYP7B1	−761,491	ARMC1	73,633	0
chr7	29,605,808	29,606,350	4	2.04E−06	1.58E−03	WIPF3	–268,262	PRR15	2652	0
chr17	80,195,101	80,195,403	3	1.51E−06	2.09E−03	SLC16A3	3689	CSNK1D	36,355	0
chr22	43,739,992	43,740,231	3	1.48E−06	2.59E–03	SCUBE1	−718			0
chr4	3,365,280	3,365,443	4	2.12E−06	5.44E−03	HGFAC	–78,252	RGS12	49,488	0
chr17	81,028,481	81,028,497	2	3.24E−07	8.47E–03	B3GNTL1	–18,803	METRNL	−9078	0
chr2	3,699,195	3,699,354	4	5.24E−06	1.37E−02	ALLC	−6510	COLEC11	49,779	0
chr22	43,525,330	43,525,432	2	3.86E−06	1.58E−02	MCAT	14,019	BIK	18,627	0
chr22	38,506,589	38,506,782	4	1.09E−05	2.34E−02	BAIAP2L2	−9			0
chr2	236,100,688	236,100,752	3	4.77E−06	3.08E−02	AGAP1	−302,031	SH3BP4	213,391	0
chr7	155,150,681	155,150,794	2	9.95E−06	3.63E−02	EN2	−100,086	INSIG1	61,252	0

Bold values represent 3 DMRs taken forward for further analyses because, in addition to being identified from the meta-analysis, they were also identified in at least two cohorts, when analyses were run in each cohort separately.

### Effects of differential methylation on gene expression

**Fig. 2:**
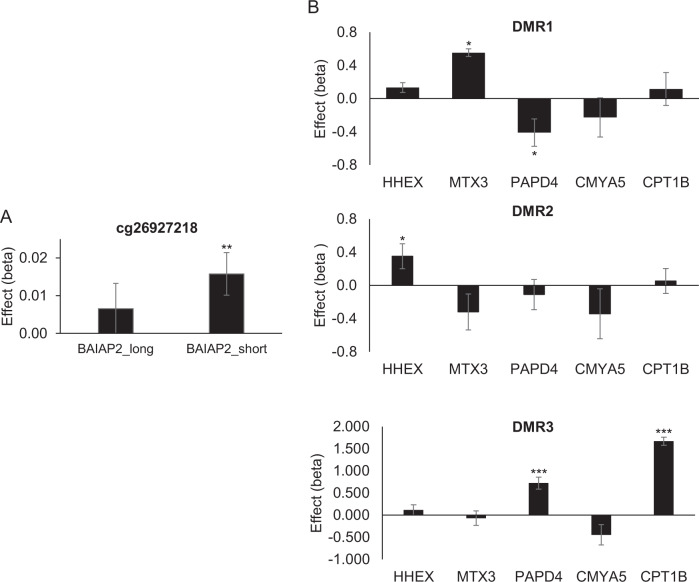
Analyses of top CpG (**a**) and DMRs (**b**) demonstrate effects of DNA methylation on gene expression in 631 subjects from the IMAGEN sample. In the DMR analyses, linear regression analyses tested relationship between methylation at the listed DMR and expression of *HHEX*, *MTX3, PAPD4, CMYA5* and *CPT1B*, controlling for methylation at the other two DMRs. Results represent unstandardized coefficients ± S.E.M. *p < 0.05; **p < 0.01; ***p < 0.001

We measured the impact of DNA methylation on expression of neighbouring genes (*cis*-effects) in 631 IMAGEN subjects for which DNA methylation and mRNA expression data were available (see Supplementary Materials and Methods). Methylation at most loci affected gene expression, with the effects of DMRs being larger than that of individual CpGs (i.e. cg26927218). Several isoforms are expressed from *BAIAP2*, and isoform-specific effects were observed for cg26927218; methylation at this locus correlated with increased expression of the short isoform for *BAIAP2* (*β* = 0.016, *P* = 5 × 10^−3^; Fig. 2a). There were no significant effects of cg17858098 on *ECH1* mRNA levels (*β* = −0.008, *P* = 0.201). Given the correlations between the selected three DMRs noted above, we controlled for methylation at the other two DMRs when testing for effects of a given DMR on gene expression. As shown in Fig. 2b, DMR1 methylation had no effect on expression of *CMYA5* (*β* = −0.227, *P* = 0.492), tending instead to have contrasting effects on expression of neighbouring genes (*β* = −0.410, *P* = 0.039 and *β* = 0.554, *P* = 0.019 for *PAPD4* and *MTX3*, respectively). Methylation at DMR2 increased expression of its closest gene, *HHEX* (*β* = 0.351, *P* = 0.020). Methylation at DMR3 had strong effects on expression of the adjacent *CPT1B* gene (*β* = 1.670, *P* = 2.55 × 10^−59^). *Trans-*effects were also noted for this DMR, as it associated with increased expression of *PAPD4* (*β* = 0.724, *P* = 1.21 × 10^−7^), a gene adjacent to DMR1.

### Correlations of DNA methylation between blood and brain

To investigate if the above findings would remain relevant for the brain, we first compared methylation levels at the selected differentially methylated loci (i.e. two CpG sites and three DMRs) in blood and brain tissues sampled from the same individuals to establish the degree to which blood methylation levels at selected loci correlated with their brain methylation patterns. Then, we compared the degree of these blood–brain covariations (i.e. the extent to which of DNA methylation in blood correlated with DNA methylation in brain) to the corresponding *Z*-values from the hippocampal EWAS. We evaluated these effects across all three DMRs, as well as within each DMR. It is important to point out that higher degree of blood–brain covariations in methylation, which indicates a higher proportion of shared information between blood and brain, would result in increased strength in association between blood DNA methylation and hippocampus volume, solely if this association was indeed mediated by brain DNA methylation. Please see Supplementary Materials and Methods for details of the approach.

We compared methylation levels at these sites in blood and brain tissues (prefrontal cortex, entorhinal cortex, superior temporal gyrus and cerebellum) sampled from the same individuals (*N* = 75) using the blood–brain DNA methylation comparison tool [56] (see Supplementary Materials and Methods; Supplementary Table 3). There was no significant correlation between blood and brain methylation levels at the individual CpG sites (cg26927218—*BAIAP2* and cg17858098—*ECH1*). On the other hand, interindividual variation in whole blood was a moderate predictor of interindividual variation in all tested cortical brain areas for DMR1 and DMR3 (strongest correlations: *r* = 0.54, *P* = 1.20 × 10^−6^ and *r* = 0.59, *P* = 2.37 × 10^−8^, respectively; Supplementary Table 3). For DMR2, correlations were more varied with the strongest correlation in the superior temporal gyrus (*r* = 0.37, *P* = 9.68 × 10^–4^; Supplementary Table 3). Generally, correlations were stronger in cortical brain regions than in the cerebellum. Crucially, the degree of blood–brain covariations in DNA methylation at these sites predicted associations between blood DNA methylation and hippocampus volume with moderate to large effect sizes (Supplementary Table 4 and Supplementary Fig. 1). These effects were particularly notable in the superior temporal gyrus, across all three DMRs (*r* = 0.751, *t* = 4.54, df = 16, *P*_one tailed_ = 6.6 × 10^−3^ after Bonferroni correction for four brain regions), and at each DMR (*r* *=* 0.707, *t* = 2.65, df = 7, *P*_one tailed_ = 0.017 for DMR1; *r* *=* 0.964, *t* = 5.14, df = 2, *P*_one tailed_ = 0.018 for DMR2; *r* *=* −0.748, *t* = −2.52, df = 5, *P*_one tailed_ = 0.027 for DMR3). There was also moderate prediction by the degree of covariation of DNA methylation in prefrontal cortex, across all three DMRs (*r* = 0.417, *t* = 1.84, df = 16, *P*_one tailed_ *=* 0.042), and with DMR2 (*r* = 0.966, *t* = 5.26, df = 2, *P*_one tailed_ *=* 0.017). These results strongly suggest that associations between hippocampal volume and blood DNA methylation levels at the selected DMRs are largely mediated by their DNA methylation levels in the brain (see Supplementary Materials and Methods for more details).

Another comparison between methylation in blood and other brain regions—Brodmann area (BA)7 (parietal cortex); BA10 (anterior prefrontal cortex) and BA20 (ventral temporal cortex)—using a smaller dataset of 16 BECon post-mortem samples [57] revealed similar patterns (see Supplementary Materials and Methods; Supplementary Fig. 2). For DMR1, there were moderate correlations between blood and BA7 methylation at all CpGs (*r* = 0.13–0.47) and between blood and BA10 for most CpGs (*r* = 0.13–0.30). For DMR3, correlations between blood and brain methylation were strong in all areas (*r* = 0.37–0.86), while the degree of correlations varied at DMR2 ranging from −0.35 to 0.34, depending on the CpG site and the brain area.

### Genetic contributions to differential DNA methylation associated with hippocampal volume

Given that genetic factors may underlie the correlations between DNA methylation in different tissues, we searched for methylation QTLs in two datasets. A search in the ARIES mQTL database [58] identified several SNPs associated with methylation at the DMR1 and DMR3 loci (see Supplementary Materials and Methods; Supplementary Table 5A). The strongest mQTLs, rs131758 and rs4441859, affected methylation such that the A-allele at these SNPs associated with increased methylation at DMR3 and DMR1, respectively. These effects were replicated in two other datasets [59, 60] (see Supplementary Materials and Methods; Supplementary Table 5B). Remarkably, eQTL analyses indicated that these alleles correlated with expressions of *CMYA5* and *CPT1B*, albeit differently. While the effects of the rs4441859_A allele were tissue-specific, the rs131758_A allele increased *CPT1B* expression in all tissues, including the brain (Supplementary Table 5C and Supplementary Fig. 3).

Furthermore, we considered whether there was a significant overlap between DNA methylation differences identified in this study and SNPs associated with hippocampal volume. To test this, we used the recent genome-wide association studies of hippocampal volume conducted by ENIGMA [18] (excluding the IMAGEN data; GWAS association thresholds *P* < 5 × 10^–6^ and *P* < 5 × 10^−7^) as a dataset for significant hippocampal SNP regions, adapting MAGENTA [61] (see Supplementary Materials and Methods), the gene sets-based enrichment analysis tool for GWAS data to the analysis of methylation data. SNPs were merged into genomic regions that were then examined for overlap with DNA methylation identified in hippocampal EWAS performed in the IMAGEN sample. These analyses revealed a significant overlap between DNA methylation loci and SNP loci influencing hippocampal volume (Supplementary Table 6).

## Discussion

In this large epigenome-wide meta-analysis we identified for the first time differentially methylated CpG sites and genomic regions whose levels of DNA methylation correlate with variation in hippocampal volume. We further demonstrate the potential of using blood to discover epigenetic biomarkers for the living human brain. Methylation at these sites affect the expression of genes required for hippocampal function and metabolic regulation. At the identified sites, the observation that DNA methylation variation in blood can mirror that of brain tissues, and that the degree of this covariation could predict the association of blood DNA methylation with hippocampus volume, helps us generate hypotheses as to how modifiable factors such as diet and lifestyle may contribute to some of the impairments associated with diabetes and neurodegenerative conditions [62].

Changes in hippocampal volumes are hallmarks of brain development predictive of cognitive deficits generally associated with aging and neurodegeneration. While large hippocampal volume is linked with good memory and cognitive function, hippocampal atrophy is associated with the development of a range of neurodegenerative [63] and neuropsychiatric disorders [6–8, 10]. Modifiable factors such as obesity, exercise, stress and medication can reduce or increase the size of the hippocampus throughout life [63]. Collectively, our findings support these observations, pointing to associations of hippocampal volume with fatty acid metabolism, as discussed below.

Two of the top hits identified (*CPT1B* and *ECH1*) encode key enzymes involved in β-oxidation of fatty acids. These enzymes act on the same pathway, CPT1B being necessary for the transport of long-chain fatty acids into the mitochondria and ECH1 for a key step in their β-oxidation. Fatty acids (notably the omega-3 polyunsaturated fatty acids) benefit brain development and healthy brain aging by modulating neurogenesis and protecting from oxidative stress throughout the lifespan [64]. More specifically, neural precursors in the hippocampus and subventricular zone require fatty acid oxidation for proliferation [65]. This led to the proposition that abnormalities in brain lipid metabolism contribute to hippocampal dysfunction in AD by their ability to suppress neurogenesis at early stages of disease pathogenesis [66]. Accordingly, fatty acid metabolism in the brain seems to be closely related to the pathogenesis of Alzheimer’s disease [67].

Further links between metabolism and hippocampal volume were suggested by our identification of a region annotated to a replicated risk locus for T2D (*HHEX*) [53]. The metabolic alterations observed in T2D may induce cognitive dysfunction [68] by exacerbating declines in hippocampal volumes associated with aging [69] and AD pathology [70], a process to which *HHEX* may contribute [71]. This is supported by findings that genetic variations within the *HHEX* gene region may underlie the association of T2D with AD, with the *HHEX* rs1544210_AA genotype interacting with diabetes to increase the risk of dementia and AD by more than fourfold [71]. Furthermore, individuals with diabetes who carry the *HHEX* rs1544210_AA genotype tend to have significantly smaller hippocampal volumes than those without these conditions [71].

DNA methylation at most loci had clear, albeit distinct effects on gene expression. Notable transcript-specific effects were observed for cg26927218 on *BAIAP2*. The cg26927218 locus is located in a DNase I hypersensitive site, characteristic of regions actively involved in transcriptional regulation [72], within a consensus DNA binding sequence for the MYC associated factor X (MAX)—a transcription factor controlling cell proliferation, differentiation, and apoptosis. MAX belongs to a class of transcription factors that recognize CpG-containing DNA binding sequences, only in their unmethylated form [73, 74]. Thus, methylation at cg26927218 may affect expression of the *BAIAP2* short variant by directly interfering with the function of this transcription factor. A role for the region surrounding cg26927218 in transcriptional regulation is further supported by findings showing that a genetic variant (rs8070741) near cg26927218 enhances cortical expression of the *BAIAP2* short variant [75].

Besides the hippocampus, none of the other two subcortical structures investigated generated significant results. This may reflect a unique role of the hippocampus in brain development, possibly related to it being a site of neurogenesis. These findings are also consistent with the relative heritability of the different subcortical structures, indicating higher twin-based heritability estimates for larger (hippocampus and thalamus) compared with smaller (NAcc) subcortical structures but overall low SNP-based heritability [18]. This supports the model according to which a substantial fraction of the heritability of complex traits is due to epigenetic variation [34]. Our analyses on genetic contributions to DMRs’ effects also suggest that epigenetic control is partially modulated by genetic variations, which is further suggested by the overlap between GWAS and EWAS of hippocampal volume.

In conclusion, we have identified DNA methylation at several loci that correlate with hippocampus volume, which suggest for the first time possible biological pathways by which modifiable and metabolic factors might contribute to the pathology of neurodegenerative diseases. A clear limitation is the small number of cohorts for which both MRI and DNA methylation data are available, we nonetheless provide a rigorous roadmap that should encourage larger and more extensive future studies. We also acknowledge several other limitations, such as the shortage of datasets enabling direct comparison of blood and brain DNA methylation patterns. In particular, the lack of datasets including the hippocampus as a brain region prevented us from establishing a direct link between DNA methylation in blood and in the hippocampus. However, we do provide evidence showing that DNA methylation in the superior temporal gyrus mediates associations between blood DNA methylation and hippocampal volume. Also, given the cross-sectional nature of this study, none of the associations identified can be claimed to be causal. Nevertheless, our work demonstrates the usefulness of combining peripheral DNA methylation markers and neuroimaging measures for biomarker discovery in common neurological and neuropsychiatric conditions.

## Supplementary information

Supplementary Materials and Methods

Supplementary Figures

Supplementary Table 1

Supplementary Table 2

Supplementary Table 3

Supplementary Table 4

Supplementary Table 5A

Supplementary Table 5B

Supplementary Table 5C

Supplementary Table 6

## Data Availability

The protocols used for testing association and meta-analysis and the meta-analytic results will be freely available from the ENIGMA consortium webpage upon publication (http://enigma.ini.usc.edu/protocols/ and http://enigma.ini.usc.edu/research/downloadenigma-gwas-results).
