# Supplementary material for: Epigenome-wide meta-analysis of blood DNA methylation and its association with subcortical volumes: findings from the ENIGMA Epigenetics Working Group
Source: Mol Psychiatry. 2019 Dec 6;26(8):3884–95. doi: 10.1038/s41380-019-0605-z (PMC8550939; doi:10.1038/s41380-019-0605-z)
Supplement: Supplementary file 1 — Supplementary Materials and Methods [file 41380_2019_605_MOESM1_ESM.pdf]

SUPPLEMENTARY MATERIAL AND METHODS

Subjects and brain measures

The brain phenotypes examined in this study are from the ENIGMA analysis of high-resolution MRI brain scans of volumetric measures (full details in [1]). Our analyses were focussed to mean (of left and right hemisphere) volumetric measures of three subcortical areas: the hippocampus, thalamus and nucleus accumbens, selected for their link to disease, different levels of heritability, and developmental trajectories. MRI brain scans and genome-wide DNA methylation data were available for 3,337 subjects from 11 cohorts (Supplementary Table 1). All participants in all cohorts in this study gave written informed consent and sites involved obtained approval from local research ethics committees or Institutional Review Boards.

DNA methylation microarray processing and normalization

Blood DNA methylation was assessed for each study using the Illumina HumanMethylation450 (450k) microarray, which measures CpG methylation across >485,000 probes covering 99% of RefSeq gene promoters [2], following the manufacturer's protocols.

Quality control procedures and quantile normalization were performed using the *minfi* Bioconductor package in R [3]. Briefly, red and green channels

intensities were mapped to the methylated and unmethylated status, and average intensities used to check for low quality samples. Initial quality assessment of methylation data was performed using the preprocessIllumina option. Principal component analyses (PCA) were performed using the singular value decomposition method, to identify methylation outliers based on the first four components. Samples with intensities more than 3 standard deviations away from the median were considered outliers and were removed. Intensities from the sex chromosomes were used to predict sex, and samples with predicted sex different from their recorded value were removed. Samples that were initially processed in batches were merged at this stage before further preprocessing. Stratified quantile normalization was then applied across samples The data were then normalized together using the *minfi* preprocessQuantile function [4]. PCA of normalized beta values were used to control for unknown structure in the methylation data. Most cohorts estimated the cell counts for the 6 major cell types in blood (granulocytes, B cells, CD4+ T cells, CD8+ T cells, monocytes and NK cells) for each individual by implementing the estimateCellCounts function in *minfi*, which gives sample-specific estimates of cell proportions based on reference information on cell-specific methylation signatures. Other cohorts (i.e., NTR) measured cells counts directly.

Epigenome-wide association analysis

Epigenome-wide association studies with volumes of the thalamus, hippocampus and NAcc were performed for each site separately. After normalization, probes on the sex chromosomes were filtered out (which are more difficult to accurately normalize), as were probes not detected (detection p-value > 0.01) in more than 20% of samples and probes containing a SNP (minor allele frequency ≥ 0.05) at the CpG or at the single nucleotide extension site.

We modelled association of DNA methylation and mean brain volumes in the hippocampus, thalamus and NAcc using linear regression analyses. Control variables included sex, age, age ^{2}, intracranial volume, methylation composition (the first 4 principal components of the methylation data), and blood cell-type composition (first two components of estimated cell-type proportion) and depending on the sample and disease status (when applicable). For studies with data collected across several centres, dummy-coded covariates were also included in the model. Cohorts with family data (NTR, QTIM) performed association analyses using generalized estimating equation to control for familial relationship in addition to the other covariates. Our analyses focused on the full set of subjects, including patients, to maximise the power to detect effects. We also re-analysed the data excluding patients to ensure that the effects detected were not driven by disease.

The EWAS results from each site were uploaded to a central server for meta-analyses. Cross-reactive probes were further removed from the EWAS result files from each site, leaving 397,164 probes for subsequent analysis. Results from each cohort were meta-analysed by combining correlations across all 11 cohorts with fixed effect model, weighting for sample size [5]. False discovery rates (FDR) were computed (correcting for the number of brain regions tested and the number of DNA methylation probes) and FDR < 0.05 was considered statistically significant. The protocols used for testing association and meta-analysis and the meta-analytic results will be freely available from the ENIGMA consortium webpage upon publication (http://enigma.ini.usc.edu/protocols/ and http://enigma.ini.usc.edu/research/downloadenigma-gwas-results).

Identification of differentially methylated regions (DMRs)

We identified DMRs by applying the *Comb-p* algorithm [6] on the meta-analysis of hippocampal volume. *Comb-p* adjusts *p*-values for genomic autocorrelation (ACF), identifies enriched regions of low *p*-values, and performs inference on putative DMRs using Sidăk multiple testing correction [7]. The ACF distance was set to 500bp and the *p*-value threshold required for a DMR at $p < 0.05$. DMRs contained a minimum of 2 CpG sites.

Effects of methylation on gene expression

Effects of DNA methylation on gene expression were investigated in 631 subjects of the IMAGEN sample for which gene expression data were available. Total RNA was extracted from whole blood cells collected at the age of 14 using the PAXgene Blood RNA Kit (QIAGEN Inc., Valencia, CA). Following quality control of the total RNA extracted, labeled complementary RNA (cRNA) was generated using the Illumina® TotalPrep™ RNA Amplification kit (Applied Biosystems/Ambion, Austin, TX). The size distribution of cRNA was determined through Bioanalyzer (Agilent Technologies, Santa Clara, CA) using the Eukaryotic mRNA Assay with smear analysis. Gene expression profiling was performed using Illumina HumanHT-12 v4 Expression BeadChips (Illumina Inc., San Diego, CA). Expression data were normalized using the mloess method [8]. Expression data for genes mapping the top two CpG sites and DMRs associated with hippocampus volume. These included *BAIAP2* (probes ILMN_1705922, ILMN_1652865, ILMN_1699727, ILMN_2247226 and ILMN_2258749), *ECH1* (ILMN_1653115), *CMYA5* (ILMN_1805765) and its neighbouring genes *MTX3* (ILMN_1679071) and *PAPD4* (ILMN_1681845) genes, HHEX (ILMN_1762712) and *CPT1B* (ILMN_1791754). Expression data were log-transformed before analyses. For each DMR, a single DNA methylation factor was computed, taking into account methylation at all CpG sites within the DMR. Associations between gene expression and DNA

methylation were measured using linear regressions with the first 4 principal components of the methylation data, sample batches, the first two components of estimated cell-type proportion, recruitment centres (dummy-coded) and sex as covariates.

Methylation quantitative trait loci (mQTL)

To determine the relationship between genetic variation and CpG methylation levels, we searched for mQTLs in several datasets. First, we interrogated the ARIES dataset [9] that includes DNA methylation collected from peripheral blood (or cord blood) at five different time points across the life course from individuals in the Avon Longitudinal Study of Parents and Children (ALSPAC) [10]. This dataset applied conservative multiple testing correction ($p < 1 \times 10^{-14}$) to identify between 24,262 and 31,729 sentinel associations at each time point.

We complemented this search using data from the combined Lothian Birth Cohorts (1921 and 1936) [11], and the Brisbane Systems Genetics Study [12]. The discovery and replication thresholds set in that study were $P < 1 \times 10^{-11}$ and $P < 1 \times 10^{-6}$, respectively, with both cohorts acting as a discovery ($P < 1 \times 10^{-11}$) and replication ($P < 1 \times 10^{-6}$) data set (only the most significant SNP for each CpG was considered).

Expression quantitative trait loci (eQTL)

We used the Genotype-Tissue Expression (GTEx) database [13] to identify expression quantitative trait loci (*cis*-eQTLs; i.e., SNPs correlating with differential expression of neighbouring genes). This dataset, generated from 48 tissues from 620 donors, tests for significant SNPs-genes pairs for genes within 1Mb of input SNPs. The data described in this manuscript were obtained from the GTEx Portal (https://gtexportal.org/home/), Release: V7. It used FastQTL [14], to map SNPs to gene-level expression data and calculate q-values based on beta distribution-adjusted empirical p-values. A false discovery rate (FDR) threshold of <0.05 was applied to identify genes with a significant eQTL. The effect sizes (slopes of the linear regression) were computed in a normalized space (i.e., normalised effect size (NES)), where magnitude has no direct biological interpretation. They reflect the effects of the alternative alleles relative to the reference alleles, as reported in the GTEx database.

Brain-blood methylation correlation

We interrogated a searchable DNA methylation database [15] (https://epigenetics.essex.ac.uk/bloodbrain/) generated from matched DNA samples isolated from whole blood and 4 brain regions (prefrontal cortex, entorhinal cortex, superior temporal gyrus, and cerebellum) from 122 individuals to establish the degree to which blood methylation levels at selected loci correlated with their brain methylation patterns. Correlations between blood

and brain methylation levels at individual CpG loci within DMRs were evaluated to indicate the similarity of methylation level between blood and brain tissues at these loci. In addition, the degree of these blood-brain co-variations (i.e. the extent to which of DNA methylation in blood correlated with DNA methylation in brain) at selected CpG loci and across DMRs were compared to their corresponding Z-values from the hippocampal EWAS. This comparison enabled us to evaluate the possible transmission of information from blood DNA methylation through brain DNA methylation to hippocampus volume.

It is important to point out that higher degree of blood-brain co-variations in methylation, which indicates a higher proportion of shared information between blood and brain, would result in increased strength in association between blood DNA methylation and hippocampus volume, solely if this association was indeed mediated by brain DNA methylation. Specifically, we used the following linear models to test if associations between hippocampus volume and blood DNA methylation (as found in our EWAS) were mediated by brain DNA methylation:

$$BRM_{ij} = BS_j + \varepsilon_{ij}$$
$$BLM_{ij} = \beta_i \times BRM_{ij} + \eta_{ij}$$

where *BLM*, *BRM* and *BS* denote blood DNA methylation, brain DNA methylation and brain structure (i.e., hippocampus volume), respectively; ε_{ij} and η_{ij} the residual terms of the first and second equations of the *i*th CpG site (*i*=0,1,2,...,*m*, where *m* indicates the number of CpG sites) from the *j*th individual

(j=0,1,2,...,n, where n indicates the number of individuals) with $E_i(\varepsilon_{ij})=0$, $Var_i(\varepsilon_{ij})=\rho_{\varepsilon_i}^2>0$, $E_i(\eta_{ij})=0$, $Var_i(\eta_{ij})=\rho_{\eta_i}^2>0$ and $Cov_i(\varepsilon_{ij},\eta_{ij})=0$; β_i is the parameters for the ith pair of brain and blood CpG site. We could then derive the t-statistics at each CpG site for both correlations/associations (i.e. the brain-blood co-variations in methylation and the correlation between blood DNA methylation and hippocampus volume) as:

$$\begin{aligned} t_{BLM_i,BRM_i} &\sim \frac{Cor\left(BLM_i, BRM_i\right)}{\sqrt{1-Cor^2\left(BLM_i, BRM_i\right)}} \\ &= \frac{\beta_i\sqrt{Var\left(BS\right)+Var\left(\varepsilon_i\right)}}{\sqrt{Var\left(\eta_i\right)}} \\ &= \frac{\beta_i\sqrt{\rho_{BS}^2+\rho_{\varepsilon_i}^2}}{\rho_{\eta_i}} \end{aligned}$$

and

$$\begin{aligned} t_{BLM_i,BS} &\sim \frac{Cor\left(BLM_i, BS\right)}{\sqrt{1-Cor^2\left(BLM_i, BS\right)}} \\ &= \frac{\beta_i\sqrt{Var\left(BS\right)}}{\sqrt{\beta_i^2 Var\left(\varepsilon_i\right)+Var\left(\eta_i\right)}} \\ &= \frac{\beta_i\rho_{BS}}{\sqrt{\beta_i^2\rho_{\varepsilon_i}^2+\rho_{\eta_i}^2}} \end{aligned}$$

, respectively, where ρ_A denotes the standard deviation of variable A, i.e. one of BS, ε_i and η_i. If each of the two t-statistics shown above was observed from an independent sample, we could have:

$$
\begin{aligned}
Cor\left(t_{BLM_i,BRM_i}, t'_{BLM_i,BS}\right) &= E\left(\frac{\beta_i\sqrt{\rho_{BS}^2+\rho_{\varepsilon_i}^2}}{\rho_{\eta_i}} \times \frac{\beta'_i\rho'_{BS}}{\sqrt{\beta_i'^2\rho_{\varepsilon_i}'^2+\rho_{\eta_i}'^2}}\right) - E\left(\frac{\beta_i\sqrt{\rho_{BS}^2+\rho_{\varepsilon_i}^2}}{\rho_{\eta_i}}\right) \times E\left(\frac{\beta'_i\rho'_{BS}}{\sqrt{\beta_i'^2\rho_{\varepsilon_i}'^2+\rho_{\eta_i}'^2}}\right) \\
&= E\left(\frac{\rho'_{BS}\sqrt{\rho_{BS}^2+\rho_{\varepsilon_i}^2}}{\rho_{\eta_i}}\right) \times E\left(\frac{\beta_i\beta'_i}{\sqrt{\beta_i'^2\rho_{\varepsilon_i}'^2+\rho_{\eta_i}'^2}}\right) - E\left(\frac{\rho'_{BS}\sqrt{\rho_{BS}^2+\rho_{\varepsilon_i}^2}}{\rho_{\eta_i}}\right) \times E\left(\beta_i\right) \times E\left(\frac{\beta'_i}{\sqrt{\beta_i'^2\rho_{\varepsilon_i}'^2+\rho_{\eta_i}'^2}}\right) \\
&= E\left(\frac{\rho'_{BS}\sqrt{\rho_{BS}^2+\rho_{\varepsilon_i}^2}}{\rho_{\eta_i}}\right) \times \left[E\left(\frac{\beta_i\beta'_i}{\sqrt{\beta_i'^2\rho_{\varepsilon_i}'^2+\rho_{\eta_i}'^2}}\right) - E\left(\beta_i\right) \times E\left(\frac{\beta'_i}{\sqrt{\beta_i'^2\rho_{\varepsilon_i}'^2+\rho_{\eta_i}'^2}}\right)\right] \\
&= E\left(\frac{\rho'_{BS}\sqrt{\rho_{BS}^2+\rho_{\varepsilon_i}^2}}{\rho_{\eta_i}}\right) \times Cor\left(\beta_i, \frac{\beta'_i}{\sqrt{\beta_i'^2\rho_{\varepsilon_i}'^2+\rho_{\eta_i}'^2}}\right)
\end{aligned}
$$

, where $\rho_{\varepsilon_i}^2$, ρ_{η_i}, $\rho_{\varepsilon_i}'^2$ and $\rho_{\eta_i}'^2$ were assumed to be independent from each other. Clearly, a null result of $Cor\left(t_{BLM_i,BRM_i}, t'_{BLM_i,BS}\right)$ could be achieved if at least one of the two terms on the right-hand side of the last equation equals 0. While the first term can hardly reach 0 unless ρ'_{BS} equals 0, indicating the brain structure is identical among individuals from the second sample, and hence no transmission of information between the brain methylation and the brain structure (i.e. their correlation equals 0 at each methylation site), the second term reaches 0 only if β_i is independent from β'_i, i.e. the transmissions of information between blood and brain DNA methylations (i.e. the blood-brain co-variation) in the two samples are completely different. Therefore, a significant statistical test supporting $Cor\left(t_{BLM_i,BRM_i}, t'_{BLM_i,BS}\right) > 0$ should indeed suggest a mediation role of DNA methylation in the proposed brain areas on the transmission of information between the blood DNA methylation and the brain structure of interest. Note no transmission direction (e.g. from blood DNA methylation to brain structure) could be derived from this approach.

We evaluated this transmission effect across all three DMRs, as well as within each DMR. When all three DMRs were analysed together, dummy variables were introduced to control for varied blood-brain correlation levels at each DMR. For each DMR, as the direction of transmission effect was expected to follow that of the EWAS, we applied one-tailed statistical tests (i.e. tests for the right tail in DMR1 and DMR2 and a test for the left tail in DMR3). Also of note, when investigating effects across all three DMRs, we reversed the sign of Z-values in DMR3 to harmonize the expected direction of associations.

An additional search was performed using data from blood and Brodmann areas 7, 10 and 20 from post-mortem samples of 16 individuals [16].

Enrichment analyses

To test for enrichment for genomic regions found associated with hippocampal volume in our recent GWAS meta-analysis of hippocampal volume [1], we performed analyses based on MAGENTA [17], a computational tool designed for gene sets-based enrichment analyses with GWAS meta-analyses data as an input. To avoid “double dipping” in these analyses, we excluded the IMAGEN sample from the ENIGMA hippocampal volume meta-analysis, which we used as a dataset for known hippocampal volume SNPs (i.e., the ‘gene set’). We then tested for enrichment of this ‘gene set’ in the IMAGEN hippocampus EWAS results.

We modified the MAGENTA program to make it suitable for the analysis of DNA methylation data by first creating a ‘gene set’ of SNP regions by mapping SNPs to genomic locations, taking into account recombination hotspots. Adjacent regions with recombination rates lower than 10 were merged together. We then mapped CpG sites identified in the EWAS onto genomic regions if they fell within 100 kb of regions’ boundaries. Regions were scored based on p-values of the most significant CpG in the region. In addition, Šidák correction [7] was applied to correct for confounders such as gene size. Regions with significant enrichment were identified by permutation testing, using 5000 permutations. Two parameters were set to test for significant enrichment: *i*) the p-value threshold for selecting significant regions from the GWAS meta-analysis (GWAS thresholds of 5×10^{-6} and 5×10^{-7} were used) and *ii*) the cut-off threshold for each permutation: 90% and 99% cut-offs were used.

ACKNOWLEDGEMENTS

ENIGMA: The study was supported in part by grant U54 EB020403 from the NIH Big Data to Knowledge (BD2K) Initiative, a cross-NIH partnership, and by NIH grant R56 AG058854 to the ENIGMA World Aging Center.

IMAGEN: This work received support from the following sources: the European Union-funded FP6 Integrated Project IMAGEN (Reinforcement-related

behaviour in normal brain function and psychopathology) (LSHM-CT- 2007-037286), the Medical Research Foundation and Medical research council (grant MR/R00465X/1), the Horizon 2020 funded ERC Advanced Grant ‘STRATIFY’ (Brain network based stratification of reinforcement-related disorders) (695313), ERANID (Understanding the Interplay between Cultural, Biological and Subjective Factors in Drug Use Pathways) (PR-ST-0416-10004), BRIDGET (JPND: BRain Imaging, cognition Dementia and next generation GEnomics) (MR/N027558/1), the FP7 projects MATRICS (603016), the Innovative Medicine Initiative Project EU-AIMS (115300-2), the Medical Research Council Grant ‘c-VEDA’ (Consortium on Vulnerability to Externalizing Disorders and Addictions) (MR/N000390/1), the National Institute for Health Research (NIHR) Biomedical Research Centre at South London and Maudsley NHS Foundation Trust and King’s College London, the Bundesministeriumfür Bildung und Forschung (BMBF grants 01GS08152; 01EV0711; eMED SysAlc01ZX1311A; Forschungsnetz AERIAL 01EE1406A, 01EE1406B), the Deutsche Forschungsgemeinschaft (DFG grants, SM 80/7-2, SFB 940/2, NE 1383/14-1) and by NIH Consortium grant U54 EB020403, supported by a cross-NIH alliance that funds Big Data to Knowledge Centres of Excellence. Further support was provided by grants from: ANR (project AF12-NEUR0008-01 – WM2NA, and ANR-12-SAMA-0004), the Fondation de France, the Fondation pour la Recherche Médicale, the Mission Interministérielle de Lutte-contre-les-

Drogues-et-les-Conduites-Addictives (MILDECA), the Fondation pour la Recherche Médicale (DPA20140629802), the Fondation de l'Avenir, Paris Sud University IDEX 2012; the National Institutes of Health, Science Foundation Ireland (16/ERCD/3797), U.S.A. (Axon, Testosterone and Mental Health during Adolescence; RO1 MH085772-01A1), the Swedish Research Council (Vetenskapsrådet), the Swedish Research Council for Health, Working Life and Welfare (FORTE), the Swedish Research Council FORMAS (grant number 259-2012-23), the 111 Project (NO.B18015), the NSFC (81801773), the key project of Shanghai Science & Technology (No.16JC1420402), the Shanghai Municipal Science and Technology Major Project (No.2018SHZDZX01), ZHANGJIANG LAB, and the Shanghai Pujiang Project (18PJ1400900).

LBC1936: We thank the cohort participants and team members who contributed to these studies. Phenotype collection in the Lothian Birth Cohort 1936 was supported by Age UK (The Disconnected Mind project). Methylation typing was supported by the Centre for Cognitive Ageing and Cognitive Epidemiology (Pilot Fund award), Age UK, The Wellcome Trust Institutional Strategic Support Fund, The University of Edinburgh, and The University of Queensland. Analysis of the brain images was funded by the Medical Research Council Grants G1001401, 8200, and MR/M01311/1. The imaging was performed at the Brain Research Imaging Centre, The University of Edinburgh

(http://www.bric.ed.ac.uk), a centre in the SINAPSE Collaboration (http://www.sinapse.ac.uk). The work was undertaken by The University of Edinburgh Centre for Cognitive Ageing and Cognitive Epidemiology (http://www.ccace.ed.ac.uk), part of the cross council Lifelong Health and Wellbeing Initiative (Ref. G0700704/84698); Funding from the Biotechnology and Biological Sciences Research Council and Medical Research Council (MR/K026992/1) are gratefully acknowledged. The Scottish Funding Council contributed support through the SINAPSE Collaboration.

MPIP: The MPIP sample comprises patients included in Munich Antidepressant Response Signature study and the Recurrent Unipolar Depression (RUD) Case-Control study. We acknowledge Rosa Schirmer, Elke Schreiter, Reinhold Borschke and Ines Eidner for image acquisition and data preparation. We thank Stella Iurato for supporting quality control procedures of the methylation measurements. The MARS project was supported by the German Federal Ministry of Education and Research (BMBF) through the NGFN and NGFN-Plus programs (FKZ 01GS0481), the Molecular Diagnostics program (FKZ 01ES0811), the Research Network for Mental Diseases program (FKZ 01EE1401D) and by the Bavarian Ministry of Commerce. This work was also funded by the German Federal Ministry of Education and Research (BMBF) through the Integrated Network IntegraMent (Integrated Understanding of

Causes and Mechanisms in Mental Disorders), under the auspices of the e:Med Programme (grant # 01ZX1314J to EB).

NTR: The NTR study was supported by the Netherlands Organization for Scientific Research (NWO), MW904-61-193 (Eco de Geus & Dorret Boomsma), MaGW-nr: 400-07- 080 (Dennis van ‘t Ent), MagW 480-04-004 (Dorret Boomsma), NWO/SPI 56-464-14192 (Dorret Boomsma), the European Research Council, ERC-230374 (Dorret Boomsma), and Amsterdam Neuroscience.

OATS: We thank the OATS participants and their supporters for their time and generosity in contributing to this research. We acknowledge the contribution of the OATS Research Team (https://cheba.unsw.edu.au/project/older-australian-twins-study). OATS was supported by an Australian National Health and Medical Research Council (NHMRC)/Australian Research Council Strategic Award (ID401162) and a NHMRC Project Grant (ID1045325). This research was facilitated through Twins Research Australia, a national resource in part supported by a NHMRC Centre for Research Excellence Grant (ID: 1079102).

PAFIP: This work was supported by the Instituto de Salud Carlos III (PI14/00639 and PI14/00918), MINECO (SAF2010-20840-C02-02 and

SAF2013-46292-R) and Fundación Instituto de Investigación Marqués de Valdecilla (NCT0235832 and NCT02534363). No pharmaceutical company has financially supported the study.

QTIM: Acknowledgements: We thank the twins and singleton siblings who gave generously of their time to participate in the QTIM study. We also thank the many research assistants, radiographers, and IT support staff for data acquisition and DNA sample preparation.

Funding Acknowledgements: National Institute of Child Health & Human Development (RO1 HD050735); National Institute of Biomedical Imaging and Bioengineering (Award 1U54EB020403-01, Subaward 56929223); National Health and Medical Research Council (Project Grants 496682, 1009064 and Medical Bioinformatics Genomics Proteomics Program 389891).

SYS: This work was funded by the Canadian Institutes of Health Research, Canadian Foundation for Innovation and Heart and Stroke Foundation of Canada.

UMCU: The UMCU cohort was supported by the Netherlands Organization for Health Research and Development (ZonMw) numbers 908-02-123 and 917-46-

370 (Hilleke Hulshoff Pol), and High Potential program (Hilleke Hulshoff Pol) of the Utrecht University.

REFERENCES:

1. Hibar DP, Stein JL, Renteria ME, Arias-Vasquez A, Desrivieres S, Jahanshad N *et al.* Common genetic variants influence human subcortical brain structures. *Nature* 2015; **520**(7546)**:** 224-229.

2. Sandoval J, Heyn H, Moran S, Serra-Musach J, Pujana MA, Bibikova M *et al.* Validation of a DNA methylation microarray for 450,000 CpG sites in the human genome. *Epigenetics* 2011; **6**(6)**:** 692-702.

3. Aryee MJ, Jaffe AE, Corrada-Bravo H, Ladd-Acosta C, Feinberg AP, Hansen KD *et al.* Minfi: a flexible and comprehensive Bioconductor package for the analysis of Infinium DNA methylation microarrays. *Bioinformatics* 2014; **30**(10)**:** 1363-1369.

4. Touleimat N, Tost J. Complete pipeline for Infinium((R)) Human Methylation 450K BeadChip data processing using subset quantile normalization for accurate DNA methylation estimation. *Epigenomics* 2012; **4**(3)**:** 325-341.

5. Field AP. Is the meta-analysis of correlation coefficients accurate when population correlations vary? *Psychol Methods* 2005; **10**(4)**:** 444-467.

6. Pedersen BS, Schwartz DA, Yang IV, Kechris KJ. Comb-p: software for combining, analyzing, grouping and correcting spatially correlated P-values. *Bioinformatics* 2012; **28**(22)**:** 2986-2988.

7. Sidak Z. Rectangular Confidence Regions for the Means of Multivariate Normal Distributions. *Journal of the American Statistical Association* 1967; **62:** 626-633.

8. Sasik R, Calvo E, Corbeil J. Statistical analysis of high-density oligonucleotide arrays: a multiplicative noise model. *Bioinformatics* 2002; **18**(12)**:** 1633-1640.

9. Relton CL, Gaunt T, McArdle W, Ho K, Duggirala A, Shihab H *et al.* Data Resource Profile: Accessible Resource for Integrated Epigenomic Studies (ARIES). *Int J Epidemiol* 2015; **44**(4)**:** 1181-1190.

10. Gaunt TR, Shihab HA, Hemani G, Min JL, Woodward G, Lyttleton O *et al.* Systematic identification of genetic influences on methylation across the human life course. *Genome Biol* 2016; **17:** 61.

11. Deary IJ, Gow AJ, Pattie A, Starr JM. Cohort profile: the Lothian Birth Cohorts of 1921 and 1936. *Int J Epidemiol* 2012; **41**(6)**:** 1576-1584.

12. McRae AF, Marioni RE, Shah S, Yang J, Powell JE, Harris SE *et al.* Identification of 55,000 Replicated DNA Methylation QTL. *Sci Rep* 2018; **8**(1)**:** 17605.

13. Consortium GT. The Genotype-Tissue Expression (GTEx) project. *Nat Genet* 2013; **45**(6)**:** 580-585.

14. Ongen H, Buil A, Brown AA, Dermitzakis ET, Delaneau O. Fast and efficient QTL mapper for thousands of molecular phenotypes. *Bioinformatics* 2016; **32**(10)**:** 1479-1485.

15. Hannon E, Lunnon K, Schalkwyk L, Mill J. Interindividual methylomic variation across blood, cortex, and cerebellum: implications for epigenetic studies of neurological and neuropsychiatric phenotypes. *Epigenetics* 2015; **10**(11)**:** 1024-1032.

16. Edgar RD, Jones MJ, Meaney MJ, Turecki G, Kobor MS. BECon: a tool for interpreting DNA methylation findings from blood in the context of brain. *Transl Psychiatry* 2017; **7**(8)**:** e1187.

17. Segre AV, Groop L, Mootha VK, Daly MJ, Altshuler D. Common inherited variation in mitochondrial genes is not enriched for associations with type 2 diabetes or related glycemic traits. *PLoS Genet* 2010; **6**(8).

Supplementary Figure Legends:

Supplementary Figure 1: Relationship between blood vs. brain correlation and association with hippocampal volume. The x-axis represents the effect (z-score) of individual CpGs within the listed DMR on hippocampal volume. The y-axis shows the corresponding correlation between DNA methylation in blood versus brain in 4 brain areas [56] at these CpGs. Generally, stronger effects are observed for CpG sites whose methylation levels are highly correlated in at least one tissue.

Supplementary Figure 2: Comparison between DNA methylation in blood and in three brain regions (BA7, BA10 and BA20) in paired samples from 16 individuals [57]. Metrics shown for CpG sites composing each of the 3 DMRs, include spearman correlation values of methylation between blood and the listed brain region, methylation variability in blood and brain samples and average methylation change with cell composition adjustment.

Supplementary Figure 3: Expression quantitative trait loci analyses showing effects of rs4441859 and rs131758 genotypes on *CMYA5* and *CPT1B* expression in tissues from 620 donors from the Genotype-Tissue Expression (GTEx) database [76]. Effects fulfilling the FDR threshold of ≤0.05 are highlighted in red.
