# Supplementary material for: Epigenome-wide meta-analysis of blood DNA methylation and its association with subcortical volumes: findings from the ENIGMA Epigenetics Working Group
Source: Mol Psychiatry. 2019 Dec 6;26(8):3884–95. doi: 10.1038/s41380-019-0605-z (PMC8550939; doi:10.1038/s41380-019-0605-z)

Supplementary Figure 1

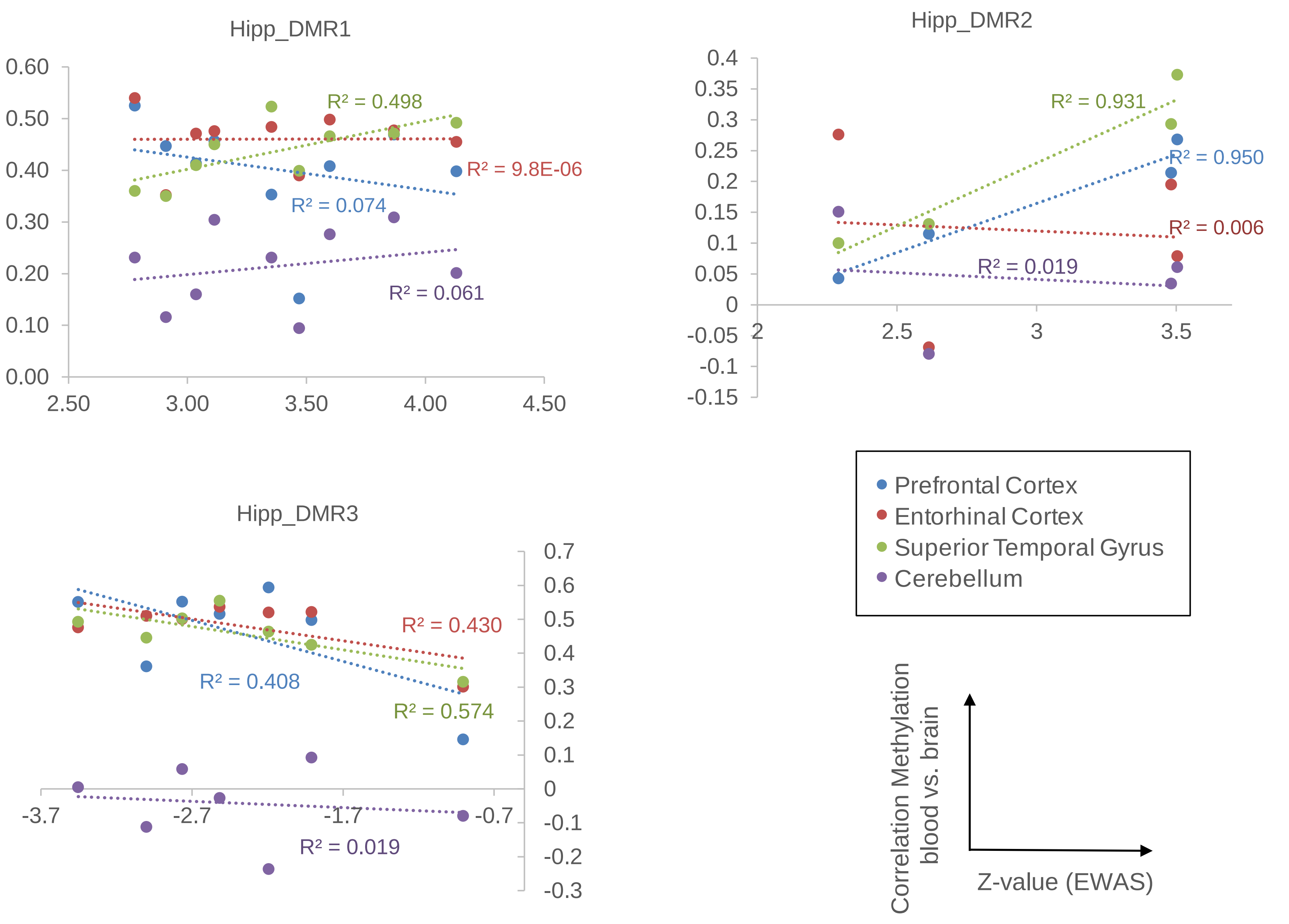

Supplementary Figure 2

	Chr	Coor	Gene(s)	Gene Region(s)	Variability BA10	Variability BA20	Variability BA7	Variability Blood	Correlation BA10	Correlation BA20	Correlation BA7	Cell Composition Blood	Cell Composition Brain
cg10299976	10	94455543	HHEX	three_plus	0.1	0.08	0.06	0.16	−0.14	−0.35	−0.05	0.03	0.01
cg14987431	10	94455650	HHEX	three_plus	0.05	0.05	0.05	0.12	0	−0.22	−0.05	0.01	0.01
cg07856667	10	94455710	None	intergenic	0.07	0.06	0.06	0.15	0.16	−0.01	0.32	0.02	0.01
cg26194475	10	94455895	None	intergenic	0.06	0.05	0.05	0.1	0.34	0.21	−0.01	0.02	0.01
cg08260245	22	51016501	CHKB−CPT1B, CPT1B, CPT1B	intragenic, intragenic, promoter	0.2	0.19	0.16	0.23	0.59	0.52	0.65	0.03	0.02
cg10490842	22	51016604	CHKB−CPT1B, CPT1B, CPT1B	intragenic, intragenic, promoter	0.13	0.12	0.09	0.23	0.73	0.58	0.69	0.03	0.01
cg19112186	22	51016638	CHKB−CPT1B, CPT1B, CPT1B	intragenic, intragenic, promoter	0.16	0.2	0.17	0.25	0.72	0.47	0.75	0.03	0.02
cg10770023	22	51016644	CHKB−CPT1B, CPT1B, CPT1B	intragenic, intragenic, promoter	0.17	0.19	0.15	0.24	0.86	0.44	0.66	0.03	0.01
cg05156901	22	51016646	CHKB−CPT1B, CPT1B, CPT1B	intragenic, intragenic, promoter	0.2	0.19	0.16	0.28	0.76	0.33	0.42	0.04	0.01
cg24363820	22	51016703	CHKB−CPT1B, CPT1B, CPT1B	intragenic, intragenic, promoter	0.17	0.16	0.12	0.26	0.76	0.39	0.59	0.03	0.01
cg00047287	22	51016899	CHKB−CPT1B, CPT1B	intragenic, promoter	0.09	0.12	0.1	0.13	0.53	0.65	0.37	0.02	0.01
cg09481121	5	78985425	CMYA5	promoter	0.11	0.11	0.07	0.15	0.19	−0.03	0.26	0.03	0.01
cg00611789	5	78985432	CMYA5	promoter	0.12	0.13	0.09	0.22	0.06	−0.19	0.33	0.05	0.01
cg11438310	5	78985434	CMYA5	promoter	0.1	0.09	0.09	0.21	0.28	−0.13	0.24	0.04	0.01
cg10257870	5	78985484	CMYA5	promoter	0.11	0.12	0.09	0.18	0.04	−0.04	0.13	0.04	0
cg03546977	5	78985489	CMYA5	promoter	0.09	0.11	0.09	0.17	0.28	0.04	0.18	0.03	0
cg09655403	5	78985495	CMYA5	promoter	0.11	0.1	0.11	0.18	−0.07	−0.01	0.17	0.04	0.01
cg15197065	5	78985562	CMYA5	promoter	0.14	0.11	0.09	0.18	0.2	−0.06	0.32	0.04	0.01
cg04154027	5	78985588	CMYA5	promoter	0.17	0.15	0.11	0.2	0.13	0.11	0.47	0.04	0.01
cg23279355	5	78985592	CMYA5	promoter	0.13	0.11	0.09	0.27	0.3	0.05	0.37	0.05	0.01

Correlation or Cell Composition Percentile or Variability Status

- 90% (Positive)
- 75−90% (Positive)
- 50−75% (Positive)
- <50% (Positive)
- <50% (Negative)
- 50−75% (Negative)
- 75−90% (Negative)
- 90% (Negative)
- 90% (Blood Cell Comp.)
- 75−90% (Blood Cell Comp.)
- 50−75% (Blood Cell Comp.)
- <50% (Blood Cell Comp.)
- 90% (Brain Cell Comp.)
- 75−90% (Brain Cell Comp.)
- 50−75% (Brain Cell Comp.)
- <50% (Brain Cell Comp.)
- Not Variable
- Variable
- Genomic Info

Supplementary Figure 3

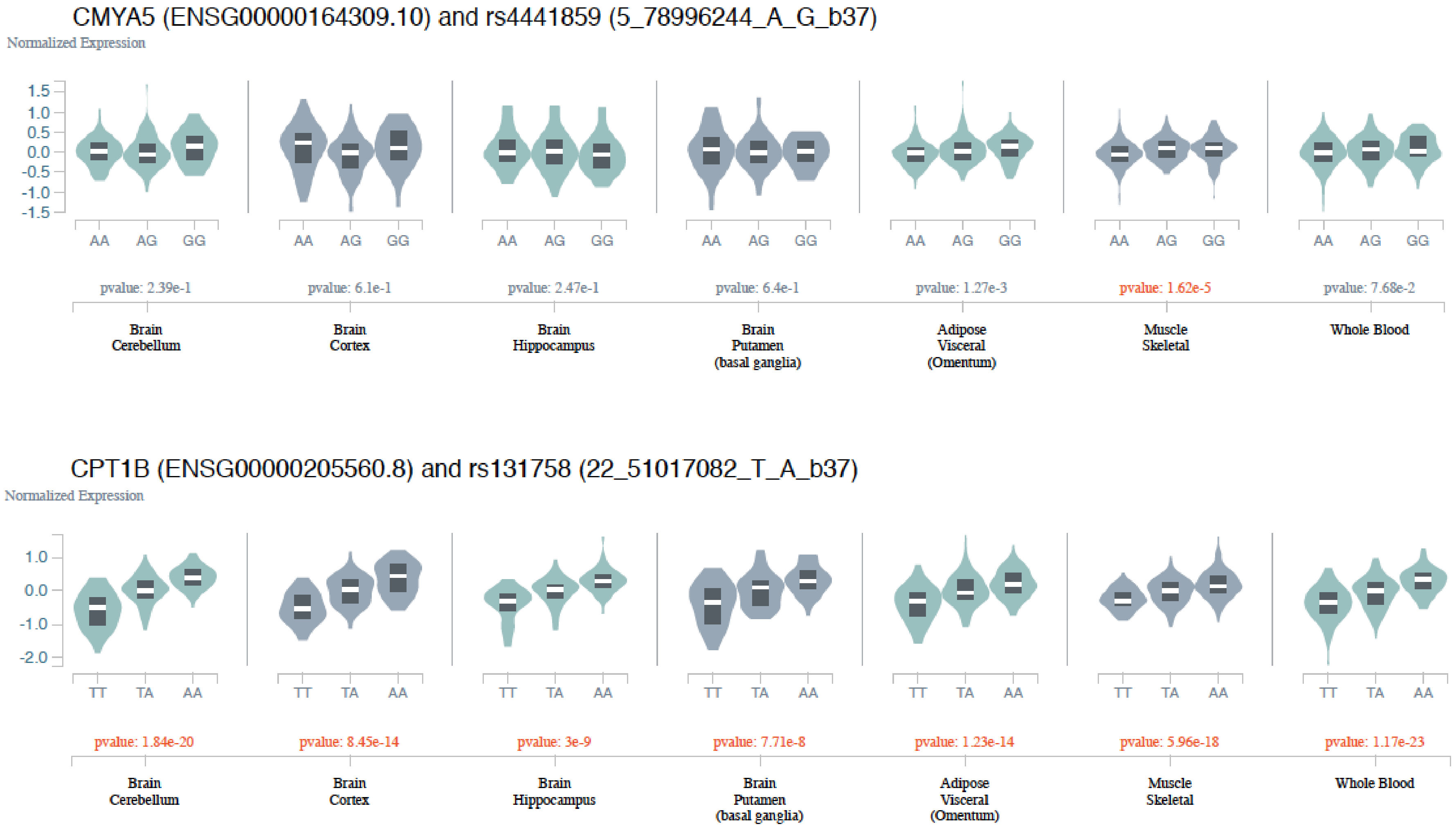

Supplement: Supplementary file 2 — Supplementary Figures [file 41380_2019_605_MOESM2_ESM.pdf]
